# Human Enriched Serum Following Hydrolysed Collagen Absorption Modulates Bone Cell Activity: from Bedside to Bench and Vice Versa

**DOI:** 10.3390/nu11061249

**Published:** 2019-05-31

**Authors:** Fabien Wauquier, Audrey Daneault, Henri Granel, Janne Prawitt, Véronique Fabien Soulé, Juliette Berger, Bruno Pereira, Jérôme Guicheux, Gael Y. Rochefort, Nathalie Meunier, Adeline Blot, Yohann Wittrant

**Affiliations:** 1 INRA, UMR 1019, UNH, CRNH Auvergne, F-63009 Clermont-Ferrand, France; Fabien_Wauquier@gmx.fr (F.W.); audrey.daneault@gmail.com (A.D.); henri.granel@inra.fr (H.G.); 2 Unité de Nutrition Humaine, Clermont Université, Université d’Auvergne, BP 10448, F-63000 Clermont-Ferrand, France; 3 Rousselot BVBA, Meulestedekaai 81, 9000 Gent, Belgium; janne.prawitt@rousselot.com; 4 Rousselot SAS, 4 rue de l’abreuvoir, 92400 Courbevoie, France; vfabiensoule@syndifrais.com; 5 CRB Auvergne, Hématologie Biologique, Equipe d’Accueil 7453 CHELTER, Centre Hospitalier Universitaire Estaing, 1 place Lucie et Raymond Aubrac, CEDEX 1, F-63003 Clermont-Ferrand, France; jberger@chu-clermontferrand.fr; 6 Biostatistics Unit (DRCI), University hospital Clermont-Ferrand, 58 rue Montalembert, 63000 Clermont-Ferrand, France; bpereira@chu-clermontferrand.fr; 7 Inserm, UMR 1229, RMeS, Regenerative Medicine and Skeleton, Université de Nantes, ONIRIS, 44000 Nantes, France; jerome.guicheux@inserm.fr (J.G.); gael.rochefort@gmail.com (G.Y.R.); 8 UFR Odontologie, Université de Nantes, 44000 Nantes, France; 9 CHU Nantes, PHU4 OTONN, 44000 Nantes, France; 10 Chu Clermont-Ferrand, Centre De Recherche En Nutrition Humaine Auvergne, 58 rue Montalembert, 63000 Clermont-Ferrand, France; nmeunier@chu-clermontferrand.fr (N.M.); ablot@chu-clermontferrand.fr (A.B.)

**Keywords:** metabolites, bone, hydrolysed collagen, nutrition, osteoporosis, absorption, collagen peptides

## Abstract

Collagen proteins are crucial components of the bone matrix. Since collagen-derived products are widely used in the food and supplement industry, one may raise the question whether collagen-enriched diets can provide benefits for the skeleton. In this study, we designed an innovative approach to investigate this question taking into account the metabolites that are formed by the digestive tract and appear in the circulation after ingestion of hydrolysed collagen. Blood samples collected in clinical and pre-clinical trials following ingestion and absorption of hydrolysed collagen were processed and applied on bone-related primary cell cultures. This original ex vivo methodology revealed that hydrolysed collagen-enriched serum had a direct impact on the behaviour of cells from both human and mouse origin that was not observed with controls (bovine serum albumin or hydrolysed casein-enriched serum). These ex vivo findings were fully in line with in vivo results obtained from a mouse model of post-menopausal osteoporosis. A significant reduction of bone loss was observed in mice supplemented with hydrolysed collagen compared to a control protein. Both the modulation of osteoblast and osteoclast activity observed upon incubation with human or mouse serum ex vivo and the attenuation of bone loss in vivo, clearly indicates that the benefits of hydrolysed collagen for osteoporosis prevention go beyond the effect of a simple protein supplementation.

## 1. Introduction

Osteoporosis is a major cause of morbidity and disability and is considered as an important contributor to medical care costs worldwide. Several treatment options are available, but concerns about possible side effects including an increased risk for cancer and cardiovascular disease have been raised [1]. Thus, prophylaxis strategies and early prevention by nutritional interventions may offer relevant alternatives [2,3,4]. The primary aim of a nutritional strategy for the prevention of osteoporosis is to provide persons at risk with a sufficient and bioavailable amount of nutrients that favours bone growth and remodelling [5]. Proteins play a major role in skeleton metabolism by providing building blocks and by exerting specific regulatory functions. Thus, protein-based supplements are promising candidates to maintain bone health during aging.

Collagen is the major structural element in the extracellular matrix of all connective tissues, including bone, and represents the most abundant protein in mammals accounting for 30% of the total protein mass in the body and 80% in the skeleton (mostly type I) [6]. In bone, collagen plays an important role in the force transmission and tissue structure maintenance [7,8]. Collagen comprises three polypeptide strands (alpha-chains) which form a unique triple-helical structure. Each strand is built by the repeating amino acid sequence Gly-X-Y, with every third amino acid being a glycine and with X and Y being mainly proline (Pro) and hydroxyproline (Hyp).

Collagen and its derivatives (gelatin and hydrolysed collagen) can be extracted from bone or skin and are widely used in the food, cosmetic and pharmaceutical industry. Hydrolysed collagen (HC), produced by enzymatic hydrolysis from gelatin, has been reported to improve joint pain and function in patients suffering from osteoarthritis [9,10,11,12,13,14], but there is a clear lack of clinical data for direct effects of HC on the skeleton. In humans, HC have mainly been used in association with other compounds such as calcium-collagen chelate, vitamin D [3,15] or curcuminoids [16] to investigate benefits on bone health, and the Group for the Respect of Ethics and Excellence in Science has comprehensively outlined that further studies are warranted to strengthen the scientific evidence, including underlying pathways, for HC effects on the bone [17].

Consistent with the growing demand for innovative tools of high physiological relevance, that can assess the efficacy of nutritional interventions, we have designed a pioneering approach taking into account metabolism at the whole body level to decipher whether and how hydrolysed collagens may exert benefits on bone tissues.

## 2. Materials and Methods 

### 2.1. Ethics

Animal models. All animal procedures were approved by the institution’s animal welfare committee (Comité d’Ethique en Matière d’Expérimentation Animale Auvergne: CEMEAA) and were conducted in accordance with the European guidelines for the care and use of laboratory animals (CE N°62–12 and 63–12).

Clinical trial. The investigations were carried out following the rules of the Declaration of Helsinki of 1975 (https://www.wma.net/what-we-do/medical-ethics/declaration-of-helsinki/), revised in 2013. The human study was approved by the French Ethical Committee [Comité de Protection des Personnes (CPP17048/N° IDRCB: 2017–A02543–50) of Saint-Germain-en-Laye - Ile de France XI]. No negative effects were reported by collagen hydrolysate ingestion (one oral dose of 25 g dissolved in 200 mL of water). The volunteers were informed of the objectives of the present study and the potential risks of ingestion of collagen hydrolysate, such as diarrhea and abdominal pain.

### 2.2. Hydrolysed Collagens 

Enzymatically hydrolysed collagens (HC) from bovine (B2000), porcine (P2000 and P5000) or fish (F2000) origin were provided by Rousselot SAS, (Courbevoie, France). Two thousand or five thousand stands for the mean molecular weight of the HC: 2kDa and 5kDa, respectively. Bovine Serum Albumin (BSA, PAA Laboratories GmbH, Austria) and hydrolysed casein (Vitalarmor-Armor Protéines, St Brice en Coglès, France) were used as a control for in vitro experiments and enriched serum production, respectively.

### 2.3. Cell Cultures

MC3T3-E1, clone 4. Murine pre-osteoblasts (MC3T3-E1, clone 4) were obtained from the American Type Culture Collection (ATCC® Number: CRL-2593™). At 80% confluence, cells cultured in 2% foetal calf serum (FCS) in the presence or absence of either BSA (0.5 mg/mL) or B2000 (0.5 mg/mL) for proliferation (seven days) and alkaline phosphatase (ALP) assays (three and seven days). For ex vivo experiments, cells were cultured in the presence of either naïve or enriched mouse serum (7.5% FCS + 2.5% mouse serum) for proliferation assays or in combination with β-glycerophosphate (5 mM) and ascorbic acid (25 µg/mL) for mineralisation assays.

Raw264.7. The murine osteoclast precursor cell line RAW264.7 was obtained from the American Type Culture Collection (ATCC® Number: TIB-71™). Cells were grown to reach 80% confluence and cultured in the presence or absence of either FCS (2%), naïve or enriched mouse serum (7.5% FCS + 2.5% mouse serum) for proliferation assays or in combination with recombinant, murine Receptor Activator of Nuclear factor Kappa-B Ligand (RANKL - 25 ng/mL) (R&D Systems) for osteoclast differentiation assays.

Primary mouse bone marrow cells. Bone marrow cells were isolated from the femur marrow cavity excised from three- to five-week-old female C3H/HeN mice. For ex vivo experiments, cells were cultured as described for RAW264.7 cells.

Human PBMC isolation and culture. Blood samples of all participants were collected in Vacutainer EDTA-containing tubes for peripheral blood mononuclear cell (PBMC) isolation. PBMCs were isolated immediately using Ficoll-Paque-Plus density-gradient centrifugation (GE Healthcare). Briefly, 15 mL of whole blood was mixed with an equal amount of PBS. The mixture was transferred to a 50 mL tube, which contained 15 mL Ficoll at the bottom. After spinning at room temperature at 300 g for 20 min, the PBMC layer was collected, washed with phosphate-buffered saline (PBS), and centrifuged to pellet the cells. Cells were then seeded at a density of 2.5 × 10^6^ cells/cm^2^ and subjected to recombinant, human RANKL (25 ng/mL) and human Monocyte Colony Stimulating Factor (M-CSF; 25 ng/mL) (R&D Systems) for osteoclast differentiation in the presence of processed naïve or enriched human serum (10%). Serum processing was performed according to the Clinic’n’Cell protocol® for cell culture optimization (DI-RV INRA #18-0058, see “Patents” section).

Primary human MSC culture. Primary human umbilical cord-derived mesenchymal stem cells (MSCs) were purchased from the American Type Culture Collection (ATCC® PCS-500-010™, Manassas, USA). Cells were grown in the presence of β-glycerophosphate (10 mM), dexamethasone and ascorbic acid (50 µg/mL) for osteoblast differentiation assays. For ex vivo experiments, cells were cultured as described above.

### 2.4. Dietary Supplementation and Ovariectomy-Related Bone Loss

In vivo model. Nine-week-old female C3H/HeN mice were purchased from JANVIER (St Berthevin, France). Mice were randomly divided into five groups (*n* = 10 per group) and housed individually for the total duration of the experiment (eight weeks). Three groups were surgically ovariectomized (OVX), two groups were sham-operated (SHAM). Mice were either given a standard diet (modified from the AIN-93M powder diet) containing 15% or 17.5% of casein or a diet containing 15% of casein + 2.5% of bovine hydrolysed collagen (B2000) resulting in the following groups: SHAM 15%; SHAM 17.5%; OVX 15%; OVX 17.5% and OVX 15% + 2.5% of B2000. The diet containing 17.5% of casein represents the isoproteic control. Diets purchased from the UPAE (Unité de Préparation des Aliments Expérimentaux – INRA Jouy en Josas, France) are described in the supplemental data section (Table S1). The experimental dose for B2000 was set to be equivalent to a dose of 10 g HC per day for a 60 kg human.

### 2.5. Tissue Sampling, Biochemical Parameters and Bone Mineral Density Analysis

Uterus atrophy was checked to ensure the efficacy of the surgically-induced estrogen deficiency, by weighing uteri at the end of the experiment. After removing all soft tissue residue, left femurs were placed in a PBS buffer with 10% formaldehyde at 4 °C. Bone mineral density was measured using an eXplore CT 120 scanner (GE Healthcare, Canada). Acquisitions were performed with X-ray tube settings at 100 kV and 50 mA. We limited our investigation to the distal trabecular region. Bone mineral density (BMD) is presented in mgHA/cm^3^. Blood samples were collected in plain tubes and centrifuged at 10000G for 5 min at room temperature. The serum was subsequently isolated, aliquoted and stored at −80 °C. OPG (osteoprotegerin) and RANKL were measured by Quantikine ELISA for mouse (R&D Systems Europe).

### 2.6. Metabolism Models and Serum Collection

Ex vivo model. Twenty-four C3H/HeN female mice (nine weeks old) were randomly divided into eight groups (for eight different time points with *n* = 3) and force-fed with 100 µL of a 50% HC solution (purified water/0.9% NaCl) corresponding to a dose of 2 g/kg body weight. Serum collection was performed under 1% isoflurane anaesthesia at 0 h, 0.5 h, 1 h, 2 h, 3 h, 6 h, 9 h and 24 h after administration to determine the maximum absorption peak. Hydroxyproline content in the serum was measured using a commercially available assay (Kit 6017; Chondrex, Inc., Redmond, Washington). In a second experiment, 30 nine-week-old female C3H/HeN mice were randomly divided into three groups (*n* = 10). Mice were force-fed with either 100 µL of purified water/0.9% NaCl (vehicle), 100 µL of a 50% hydrolysed casein solution or 100 µL of a 50% B2000 solution. Serum collection was performed under 1% isoflurane anaesthesia at 0.5 h after administration.

Human study design. A pool of 20 men (24 years old) with a mean BMI of 23.35 kg/m^2^ volunteered for this study. Volunteers were tested for blood count, renal and liver function (aspartate aminotransferase (AST), alanine aminotransferase (ALT), gamma-glutamyl transferase (GGT), urea and creatinine). Blood samples of all participants were obtained and collected in Vacutainer EDTA-containing and serum-separating tubes for PBMC isolation and serum separation, respectively. Biological samples were prepared, aliquoted and stored at the Centre de Ressources Biologiques (CRB)-Auvergne, a specialized laboratory that guarantees the quality of samples and compliance with regulatory and ethical requirements (certification according to the French standard NF S 96 900). The first step of the study aimed at determining the collagen absorption peak. Fifteen healthy volunteers, fasted for 12 h, ingested 25 g HC (B2000, P2000, P5000 or F2000; *n* = 3 per matrix) dissolved in 200 mL water. Approximately 10 mL of venous blood was collected from the cubital vein, before and every 20 min after the ingestion for a total period of 240 min. Serum was prepared from venous blood samples and stored at −80 °C until analysis. Total serum protein was quantified using the BCA kit (Sigma-Aldrich) according to the manufacturer’s protocol. Once, the absorption peak was determined, volunteers were recalled for the collection of the enriched serum fraction. Ten healthy volunteers (*n* = 10 per matrix), fasted for 12 h, ingested 25 g HC (B2000, P2000, P5000 or F2000) or 25 g of hydrolysed casein dissolved in 200 mL water. Approximately 100 mL of venous blood was drawn from the cubital vein before the ingestion of the different matrices for the isolation of PBMCs and the collection of naïve serum. At the time of the maximum absorption peak, 100 mL of blood was drawn for enriched serum production. Serum was stored at −80 °C until analysis.

### 2.7. In Vitro and Ex Vivo Assays

Cell proliferation. The cell proliferation was determined using an XTT-based method (Cell Proliferation Kit II, Sigma-Aldrich) according to the supplier’s protocol. Optical density was measured at 450 nm.

Alkaline phosphatase activity assay. ALP activity was measured after zero, three and seven days of treatment in MC3T3-E1 and zero, seven and 14 days of treatment in MSCs. Cell lysates were prepared using a Nonidet P-40 lysis buffer, p-nitrophenyl phosphate alkaline assay buffer (16.2 mM) added and the absorbance measured at 405 nm every 2 min for 30 min. Total protein was quantified to express the data as the mean OD per minute per milligram of protein.

Tartrate Resistant Acid Phosphatase (TRAP). TRAP activity was measured using p-nitrophenyl phosphate as a substrate as previously described [18]. Briefly, cell lysates were prepared using a Nonidet P-40 lysis buffer and incubated in an assay buffer (125 mm sodium acetate buffer (pH 5.2), 100 mm p-nitrophenyl phosphate (Sigma-Aldrich), and 1 mm L (+) sodium tartrate). The production of p-nitrophenol was determined at 405 nm at 37 °C and expressed as the mean absorbance/minute per milligram of protein.

Alizarin red staining. Mineralized nodules were stained with an Alizarin Red S solution (Sigma). Mineralization was evaluated by light microscopy and was quantified by the ImageJ software.

Time lapse microscopy. During videomicroscopy RAW264.7 cells were kept in a controlled environmental chamber at 37 °C, 5% CO^2^. Cell images were taken every 20 min for 96 h with the objective EC Plan-Neofluar 10x/0,3 Ph1 M27. Images were processed using the ZEN software (ZEISS, France).

Taqman Low Density Arrays (TLDA). mRNA was isolated from RAW264.7 and bone marrow cultures prior to RT (Applied Biosystems). cDNA was then subjected to TaqMan® low-density arrays (TLDAs) (Applied Biosystems 7900HT real-time PCR system). Relative expression values were calculated using the comparative threshold cycle (2−ΔΔCT) according to the Data Assist software (Applied Biosystems). 18S, GAPDH and actin served as housekeeping genes.

### 2.8. Statistical Analysis

Statistical analysis was carried out using the ExcelStat Pro software - Microsoft Office 2013, with the data expressed as means ± SD. One-way ANOVA was performed followed by a Tukey’s or T-test. Groups with significant differences (*p* < 0.05) are indicated with different letters or (*).

## 3. Results

### 3.1. Bovine HC Promotes Osteoblast Proliferation, Differentiation and Function in Vitro

**Figure 1:**
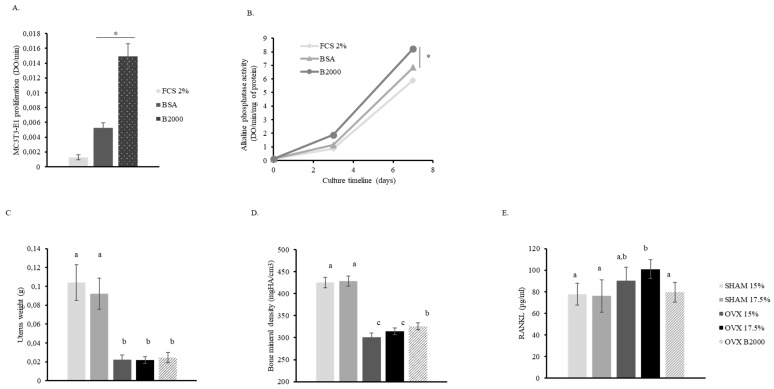
Effect of B2000 HC on bone metabolism in vitro and in vivo. (**A**) MC3T3-E1 proliferation after seven days of culture in 2% FCS (foetal calf serum) in the presence or absence of B2000 (hydrolysed collagen, 0.5 mg/mL) or its isoproteic control (BSA – bovine serum albumin: 0.5 mg/mL); (**B**) ALP activity in MC3T3-E1 pre-osteoblasts after three or seven days of culture (FCS2%; B2000: 0.5 mg/mL; BSA: 0.5 mg/mL); (**C**) uterus weight; (**D**) bone mineral density and (**E**) RANKL concentration in mouse serum. Values were obtained at the end of the in vivo experiment (eight weeks). Three groups were surgically ovariectomized (OVX), two groups were sham-operated (SH). Mice received a standard diet (modified from the AIN-93M powdered diet) containing 15% or 17.5% of casein or a diet containing 15% of casein + 2.5% of bovine HC (B2000). Groups are as follows: SHAM 15%; SHAM 17.5%; OVX 15%; OVX 17.5% and OVX B2000 (OVX15% casein + 2.5% B2000). Groups with significant differences (*p* < 0.05) are indicated with different letters (a, b, c) or (*) *p* < 0.05.

According to ethical policies for animal care, we first investigated the influence of bovine HC (B2000) on pre-osteoblast cultures in vitro. Interestingly, after seven days of culture in the presence of 0.5 mg/mL B2000, cell proliferation was three times higher than with the control protein (BSA), indicating that stimulation was likely related to a B2000 specific effect (Figure 1A). Additionally, B2000 significantly enhanced ALP activity in MC3T3-E1 cells when compared to BSA (Figure 1B).

### 3.2. B2000 Significantly Reduces Bone Loss in Vivo by Modulating the Level of RANKL

In a next step, we investigated the effect of B2000 on the bone in a preclinical model. Mice were ovariectomized to induce bone loss and mimic features of post-menopausal osteoporosis. Both uterus atrophy (Figure 1C) and subsequent bone loss (Figure 1D) were observed in ovariectomized animals, validating the experimental model. B2000 showed a slight but significant prevention of bone loss (+ 3.8%) while the control diet (casein 15%) or even the iso-proteic diet (casein 17.5%) failed to significantly counteract the OVX-induced bone alteration, supporting that, although weak, the beneficial effect of B2000 on bone loss was specific. As expected, the RANKL serum level increased upon ovariectomy. Interestingly, B2000 counteracted this rise and maintained RANKL levels at similar levels as the controls (Figure 1E), suggesting that in addition to stimulating osteoblastic function in vitro, B2000 may support an anti-osteoclastogenic effect in vivo. See supplemental data section for related in vivo data (Figures S1 and S2).

### 3.3. B2000-Enriched Mouse Serum Stimulates Osteoblast Function While Repressing Osteoclastogenesis

**Figure 2:**
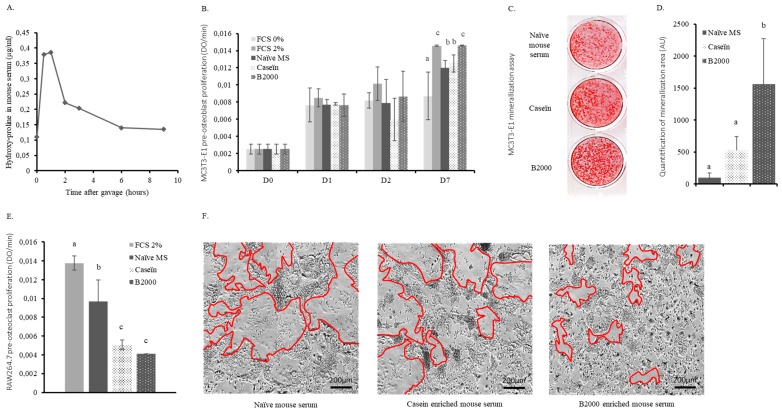
Ex vivo effects of HC in murine bone cells. (**A**) Hydroxyproline kinetics in mouse serum following B2000 (hydrolysed collagen) gavage; (**B**) MC3T3-E1 pre-osteoblast proliferation during incubation with FCS (foetal calf serum; 0 to 2%), naïve or enriched mouse serum (7.5% FCS + 2.5% mouse serum); (**C**) and (**D**) mineralisation assays and quantification. MC3T3-E1 were cultured as for proliferation assay in combination with β-glycerophosphate (5 mM) and ascorbic acid (25 µg/mL); (**E**) RAW264.7 pre-osteoclast proliferation at day four after incubation with FCS (2%), naïve or enriched mouse serum (7.5% FCS + 2.5% mouse serum); (**F**) osteoclast differentiation assays. RAW264.7 pre-osteoclast cells were cultured for four days in the presence of naïve or enriched mouse serum (7.5% FCS + 2.5% mouse serum) in combination with recombinant, murine RANKL (25 ng/mL). Red lines represent osteoclast edges and define osteoclast surface. Groups with significant differences (*p* < 0.05) are indicated with different letters (a, b, c).

To elucidate the mechanism of action and increase the physiological relevance of our approach, we set up an original ex vivo methodology taking into account the modifications that occur during the gastro-intestinal passage. Mice were forced-fed with B2000 and the absorption kinetics were recorded. As shown in Figure 2A, the hydroxyproline concentration rapidly increased in the blood reaching the absorption peak between 30 min and 60 min after ingestion. Hence, enriched serum collection was set at 45 min post-gavage with either B2000, hydrolysed casein or vehicle.

These metabolite-enriched sera were then used in cultures of MC3T3-E1 pre-osteoblasts, RAW264.7 osteoclast precursors and mouse primary bone marrow cells. MC3T3-E1 proliferation rapidly stopped in the absence of FCS (0%). After seven days of incubation, only MC3T3-E1 cells cultured in the presence of the B2000 enriched-serum (2.5%) showed a proliferation rate similar to the positive control (2% FCS). When cells were cultured with either naïve (2.5%) or hydrolysed casein-enriched serum (2.5%), proliferation was significantly lower than with the B2000-enriched serum or FCS 2% (Figure 2B). In addition, the B2000-enriched serum significantly enhanced the formation of mineralized nodules when compared to either naive or hydrolysed casein enriched serum, thus supporting an HC-dependent rather than a mere protein effect (Figure 2C,D).

On the other hand, the B2000-enriched serum significantly lowered growth of undifferentiated RAW264.7 cells (−70% compared to FCS and −58% compared to naive serum) (Figure 2E). Osteoclast differentiation was investigated and multinucleated cell formation was monitored by videomicroscopy. Interestingly, while the impact of B2000-enriched serum on RAW264.7 cell proliferation was not significantly different from hydrolysed casein-enriched serum (Figure 2E), here, in line with RANKL inhibition observed in vivo, only B2000-enriched serum inhibited the RANKL-induced giant cell formation observed after four days of differentiation (Figure 2F; osteoclast edges are marked by red lines). To further investigate the mechanism of action, both RAW264.7 and primary bone marrow cells were harvested upon enriched serum incubation for osteoclast marker expression. Consistent with the videomicroscopy data, only B2000-enriched serum significantly reduced the expression level (RQ) of osteoclast differentiation markers including cell fusion (CD36), maturation (Traf6, Csf1r and Tnfrsf1b) and activity-related genes (Acp5/TRAP and Car2) (Table 1 and Table 2). See supplemental data section for related ex vivo data (Figure S3).

### 3.4. Assays with Human Enriched Sera Confirm the in Vivo Data and Allow Comparative Activity Screening.

**Figure 3:**
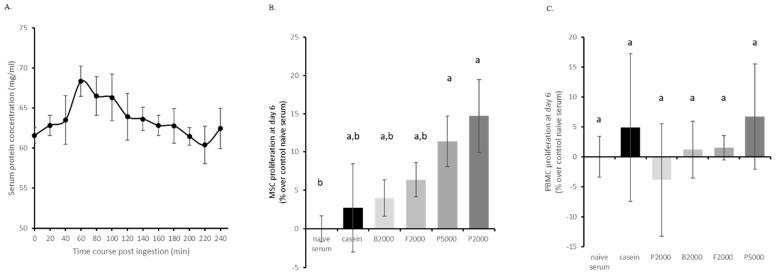
Human serum enrichment and effects on primary cell growth. (**A**) Human serum protein concentration following HC absorption; (**B**) human MSC (mesenchymal stem cells) proliferation; (**C**) human PBMC (peripheral blood mononuclear cells) proliferation. MSCs and PBMCs were cultured in the presence of 10% human enriched serum, optimized for cell culture compatibility. Naïve serum values were used for normalisation. B2000 (bovine HC; mean molecular weight 2kDa); F2000 (fish HC; mean molecular weight 2kDa); P2000 (porcine HC; mean molecular weight 2kDa) and P5000 (porcine HC; mean molecular weight 5kDa). Groups with significant differences (*p* < 0.05) are indicated with different letters (a and b).

Aiming to bring our ex vivo approach to a clinical level, we adapted our methodology to a human study. Fasted volunteers ingested 25 g of hydrolysed casein, P2000, P5000, B2000 or F2000 in 200 mL of water and the absorption kinetics were monitored as the modulation of total serum protein concentration (*n* = 10 per HC type). As shown in Figure 3A, the protein concentration rapidly increased in the blood reaching the absorption peak at 1 h after ingestion. Thus, enriched serum collection was set at 1 h post-ingestion for all treatments.

We first checked the influence of the human enriched serum on cell proliferation and viability of primary human MSCs and PBMCs as cellular models for osteoblastogenesis and osteoclastogenesis, respectively. Serum protein enrichment showed a global positive effect on MSC proliferation, however, only porcine HC exerted a significant effect compared to naïve serum (+ 11% and + 14% for P5000 and P2000, respectively) and none of the HCs were significantly different from casein (Figure 3B). Regarding cells from the hematopoietic lineage, neither casein nor any HC enriched serum had a significant effect on PBMC proliferation (Figure 3C). See supplemental data section for more detailed MSCs and PBMCs proliferation data (Figures S4 and S5).

**Figure 4:**
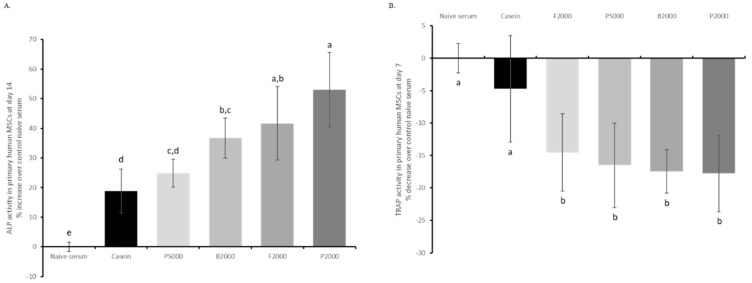
Biological activity screening of HC using human serum enrichment. (**A**) ALP (alkalin phosphatase) activity in human MSCs subjected to different human enriched sera, (**B**) TRAP (tartrate resistant acid phosphatase) activity. MSCs and PBMCs were cultured in the presence of 10% human enriched serum, optimized for cell culture compatibility. Naïve serum values were used for normalisation. B2000 (bovine HC; mean molecular weight 2kDa); F2000 (fish HC; mean molecular weight 2kDa); P2000 (porcine HC; mean molecular weight 2kDa) and P5000 (porcine HC; mean molecular weight 5kDa). Groups with significant differences (*p* < 0.05) are indicated with different letters (a, b, c, d and e).

ALP activity was assessed in human MSCs as a marker for osteoblastic commitment in the presence of the different human enriched sera. After 14 days of culture, ALP activity was significantly higher for all treatments when compared to naïve serum. Remarkably, B2000, F2000 and P2000 had the greatest effect (+36%; +41% and +53% over naïve serum, respectively) and were significantly more efficient in promoting ALP activity when compared to casein (+18% over naïve serum) (Figure 4A). In contrast, P5000 (+24% over naïve serum) failed to be different from the iso-proteic control. Regarding osteoclastogenesis, after seven days of culture all four HC enriched sera significantly inhibited RANKL-induced TRAP activity compared to both naïve and casein enriched serum (−14%, −16%, −17% and −18% for F2000, P5000, B2000 and P2000 over naïve serum, respectively) (Figure 4B). There was no significant difference between HC regarding inhibition of RANKL-induced TRAP activity in PBMCs. See supplemental data section for ALP kinetic (Figure S6).

## 4. Discussion

In this translational study we demonstrate that (1) hydrolysed collagen (HC) from bovine origin (B2000) contributes to limiting bone loss in a mouse model of post-menopausal osteoporosis. (2) Attenuation of bone loss by HC in this model was not related to the protein content of the diet but to an HC dependent effect. (3) Bone preservation by HC occurred, at least partly, through the modulation of the dialog between osteoblasts and osteoclasts (RANKL). (4) Ex vivo, mouse serum enriched with HC metabolites stimulated osteoblast activity while repressing osteoclast formation. (5) Finally, after an optimization process, human enriched serum confirmed these observations, further demonstrating the benefit of HC from other origins. Additionally, these results further validate a novel, patented clinical tool to screen potential health benefits of nutrients.

The WHO recommends a protein daily intake of around 0.8 g/kg body weight (between 50 g and 60 g of total protein for a woman of 60 kg). Current recommendations in Japan for HC supplementation range between 1 and 10 g per day (about 15% of the total daily protein intake) [19,20]. Thus, consistent with the recommendations for human supplementation the addition of HC to the animal diet was set at 15% of the total daily protein intake (corresponding to 2.5% of the diet and approximately 2.5 g/kg of body weight; Table S1. This diet was provided for eight weeks, corresponding to a 5–6 year supplementation in humans. In our study, prevention of bone loss occurred without any change in daily food intake (Figure S1A) nor diet-induced weight gain (Figure S1B–D). Therefore, the positive effect of B2000 on BMD cannot be related to bone mechanical stimulation from increased body weight. Although significant, the bone sparing effect was less important than in previous reports. Discrepancies may be due to different protocol settings. For instance, Guillerminet et al. used a diet containing 25 g/kg of body weight HC for 24 weeks, also using C3H mice, and obtained a greater bone sparing effect [21,22]. Since our experiment lasted one third of their protocol duration and we used 10 times less HC to match human recommendations, these parameters may account for the differences between the results. Although smaller, the significant prevention of bone loss in our model strongly supports the relevance of a “realistic” nutritional approach. Mouse OVX-induced osteoporosis represents a relevant tool for post-menopausal investigations. However, this acute bone loss model remains challenging for non-pharmacologic approaches. Thus, according to our data it is tempting to speculate that this HC-related nutritional strategy may be even more potent in a chronic osteoporosis model including senile osteoporosis as recently suggested in the literature [23,24,25].

The quantity of HC delivered at once and given by force-feeding to the mice was set to 2.5 g/kg of body weight to match with the in vivo experiment and allow comparison of the two methods. The quantity administered to humans was set to 0.5 g/kg of body weight. Even though this appears to be different, this dose corresponds to the one in mice when taking the metabolic weight differences between the two species into account [26]. In both mice and humans, the protein absorption peak was observed one hour after ingestion. This observation strictly correlates with available data from the literature [27,28,29,30].

Regarding the influence of B2000 on bone cell behaviour, it is worth noting that the human ex vivo data fully confirmed the observations from the mouse model. Human serum enriched with B2000 lowered osteoclastogenesis and enhanced osteoblast activity as did the mouse serum. In both cases, a modulation of cell activity was observed after HC absorption while casein had no influence. Consistent with our data, positive effects of HC on human osteoblast proliferation, differentiation and mineralized bone matrix formation were recently reported in human as well as in murine cells [31,32,33,34,35,36,37]. Furthermore, while we used a maximum of 2.5% enriched serum for the mouse cell studies, we have lately optimised the serum compatibility with our cell models to reach 10% of enriched human serum in primary human cell cultures, allowing us to omit the FCS from the medium and to further strengthen the physiological relevance of the methodology.

Recently, two trials demonstrated the clinical efficiency of collagen-derived peptides in post-menopausal women. Five grams of collagen calcium chelate containing 500 mg of elemental calcium and 200 IU vitamin D (1,25-dihydroxyvitamin D3) given daily decreased the TRAP/ALP ratio [3]. In 2018, Konig’s group reported that BMD of the spine and the femoral neck increased significantly compared to the control group when women ingested 5 g of specific collagen peptides daily [4]. Both clinical trials were run for 12 months and recruited more than a hundred volunteers to observe a bone sparing effect at statistical significance. Remarkably, the findings of Konig are fully in line with our observations. Thus, although our approach cannot fully replace a “regular” clinical trial, it may have helped the authors to secure comparable outcomes in only two months with ten times less volunteers.

The dual impact observed on both osteoblast and osteoclast cells supports the previously published data [15,21]. Interestingly, when rats received [^14^C]-Pro-Hyp orally, autoradiography revealed a noticeable cellular uptake of radioactivity in osteoblasts and osteoclasts as well as in dermal fibroblasts, epidermal cells, synovial cells and chondrocytes after 24 hours [38]. However, the biological meaning of such an accumulation of bioactive peptides in bone cells remains to be deciphered. Bone loss following the onset of menopause is mainly driven by inflammation and RANKL-induced osteoclast resorption. In vivo, we investigated the influence of hydrolysed collagen supplementation on body composition, inflammatory parameters and the release of a targeted pool of bone cytokines. Consistent with literature, ovariectomy drove adipose tissue (AT) mass gain [39,40]. However, the supplementations had no effect on this parameter supporting an AT-independent effect of hydrolysed collagen on the bone (Figure S1B–D). HC diets had no significant influence on the MCP-1 (monocyte chemotactic protein 1) circulating levels nor on the spleen weight, and the OPG serum concentration remained unchanged (Figure S2A–C). In contrast, the significant reduction of OVX-induced RANKL upregulation by B2000 may account for the observed bone-sparing effect. Cellular amino acid sensing was recently reported to occur through members of the class 3, seven transmembrane domain, G-protein receptor superfamily leading to the modulation of intracellular calcium concentration and ERK phosphorylation [41]. Since RANKL expression by osteoblasts requires ERK phosphorylation [42] and HC promotes osteoblastogenesis as well as COL1A1 expression in MC3T3-E1 osteoblasts through ERK phosphorylation, [31] these data further support the role of RANKL and MAPK signalling pathways in HC-mediated health benefits on bone. In addition, the interaction between the Asp-Gly-Glu-Ala amino acid domain of type I collagen and the α2β1 integrin receptor was proven to be an important signal for bone marrow cell differentiation towards an osteoblastic phenotype, and may contribute to our observations although we did not investigate the molecular mechanism [43].

It has been proposed that part of the HC peptides may only be digested to a certain degree within the gastrointestinal tract, with a proportion of approximately 10% of intact high molecular weight peptides reaching the blood by passing through or between enterocytes (paracellular transport and transcytosis) [9,44]. Thus, one may speculate on a differential effect of HC-derived peptides depending on size and origin. In a double-blind, placebo-controlled, randomised, clinical study on the effectiveness of HC on osteoarthritis, both HC from porcine and bovine origin were proven to be efficient for the management of osteoarthritis and the maintenance of joint health, with no reported differences [45]. However, the HC of less than 3 kDa mean molecular weight were stated to exhibit a greater osteoporosis prevention [46] or to promote bone growth in OVX- and growing rat models, respectively [47]. Accordingly, using our enriched serum methodology HC-peptides of lower molecular weight showed greater ALP stimulation in human MSCs. In contrast, the decrease of TRAP activity in RANKL-stimulated human PBMCs by HC enriched serum was not related to HC size or origin. Bone marrow and PBMC cultures comprise immune cells including lymphocytes. They are known to express RANKL and OPG that may interfere and explain the seemingly conflicting result with the RAW264.7 model [48]. Whether, the influence of HC origin and size may depend on the targeted cell type and the model remains to be elucidated.

## 5. Conclusions

Overall, our results correlate with literature data on the positive role of HC for bone health. Using a pioneering clinical screening approach, we provide further supporting evidence that the bone-sparing ability of HC is not related to a mere protein effect. We provide new insight into the mode of action showing that HC modulates osteoblast and osteoclast coupling and directly impacts bone cell activity. Thus, our results further support HC supplementation as a relevant nutritional strategy to manage bone health conditions. 

## 6. Patents

The human ex vivo methodology used in this study has been registered as a written invention disclosure by the French National Institute for Agronomic Research (INRA) (DIRV#18-0058). Clinic’n’Cell® has been registered as a trademark.

## Figures and Tables

**Table 1:** Taqman Low Density Array on differentiated RAW264.7 cells.

	CTRL (RQ)	CASEIN (RQ)	CASEIN (*p*-Value)	B2000 (RQ)	B2000 (*p*-Value)
Acp5	1.0	0.53	0.54	0.45	0.03
Car2	1.0	0.52	0.32	0.52	0.02
Csf1r	1.0	0.57	0.03	0.78	0.02
Ctsk	1.0	0.69	0.72	0.75	0.06
Itgb3	1.0	0.47	0.39	0.53	0.06
Mmp9	1.0	0.55	0.56	0.63	0.08
Nos2	1.0	0.47	0.15	0.24	0.06
Tgfbr1	1.0	0.43	0.37	0.34	0.06
Tnfrsf1b	1.0	0.58	0.17	0.49	0.07
Traf2	1.0	0.56	0.12	0.69	0.06
Traf6	1.0	0.60	0.05	0.64	0.04

CTRL: control condition; RQ: relative quantification; B2000: hydrolysed collagen 2000Da.

**Table 2:** Taqman Low Density Array on bone marrow cells.

	CASEIN (RQ)	B2000 (RQ)	B2000 (*p*-Value)
Cd36	1.0	0.66	0.04
Itgam	1.0	0.86	0.06
Tlr2	1.0	0.81	0.06
Tnfrsf1b	1.0	0.77	0.03

CTRL: control condition; RQ: relative quantification; B2000: hydrolysed collagen 2000Da.

## Supplementary Materials

The following are available online at https://www.mdpi.com/2072-6643/11/6/1249/s1, Materials: Figure S1: Murinometric parameters during the in vivo experiment. Figure S2: Inflammatory parameters and cytokine profiles. Figure S3: Alizarin red staining of the mineralization assay in MC3T3-E1 cells following enriched serum incubation (*n* = 3). Figure S4: Human primary MSC proliferation from day 1 to day 6 following enriched serum incubation. Figure S5: Human primary PBMC proliferation from day one to day six following enriched serum incubation. Figure S6: ALP activity of human primary MSCs from day 0 to day 14 following enriched serum incubation. Table S1: Composition of the diets: in vivo experiment (%).
