# Supplementary material for: Human Enriched Serum Following Hydrolysed Collagen Absorption Modulates Bone Cell Activity: from Bedside to Bench and Vice Versa
Source: Nutrients. 2019 May 31;11(6):1249. doi: 10.3390/nu11061249 (PMC6627680; doi:10.3390/nu11061249)
Supplement: Supplementary file 1 [file nutrients-11-01249-s001.pdf]

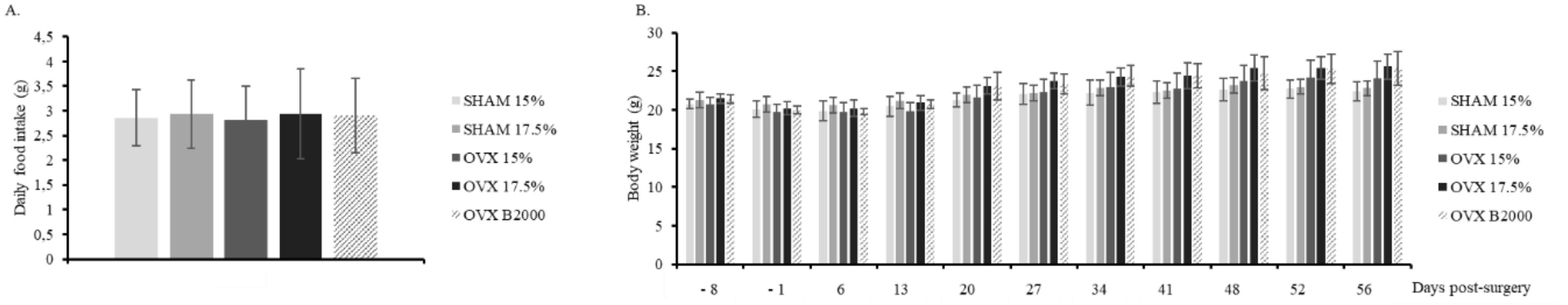

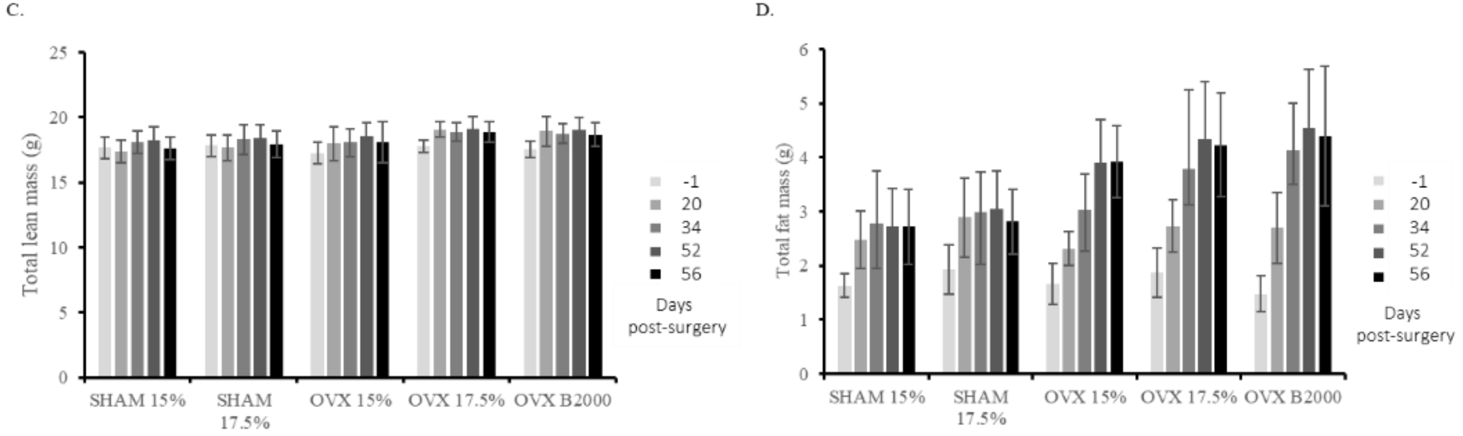

E.

Fat mass gain (g)

	ΔFat
SHAM 15%	1,085
SHAM 17.5%	0,886
OVX 15%	2,263
OVX 17.5%	2,36
OVX B2000	2,93

Figure S1. Murinometric parameters during in vivo experiment. **(A)** Daily food intake. **(B)** Body weight. **(C)** and **(D)**: Body composition as lean and fat mass, respectively. **(E)** Body fat gain during the experiment. Summary: there is no variation in daily food intake; OVX induces weight gain independently of the diet; OVX-induced weight gain relies on fat mass gain exclusively.

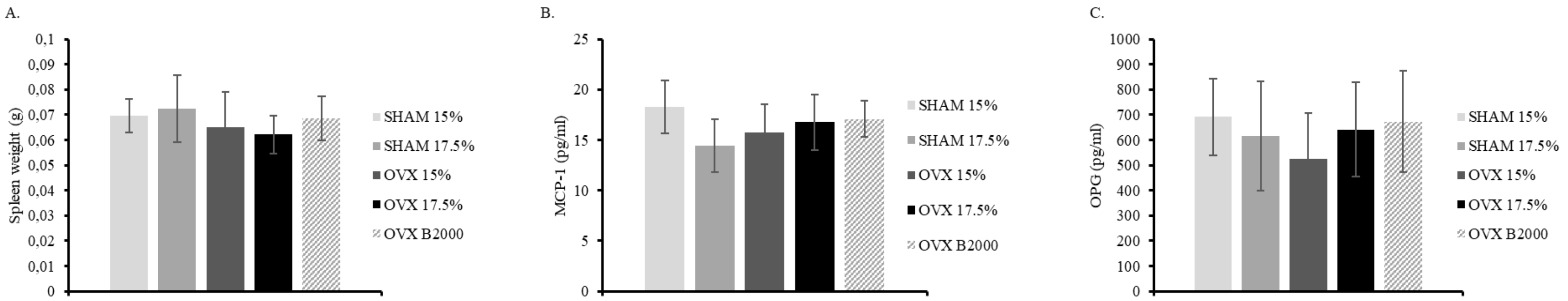

Figure S2. Inflammatory parameters and cytokine profiles. **(A)** Spleen weight. **(B)** Circulating MCP-1 serum concentration. **(C)** Circulating OPG serum concentration. Summary: there is no variation of neither spleen weight (lymphoid organ), MCP-1, nor OPG levels. The influence of HC (from bovine origin) on mouse skeleton is not related to the modulation of inflammatory parameters.

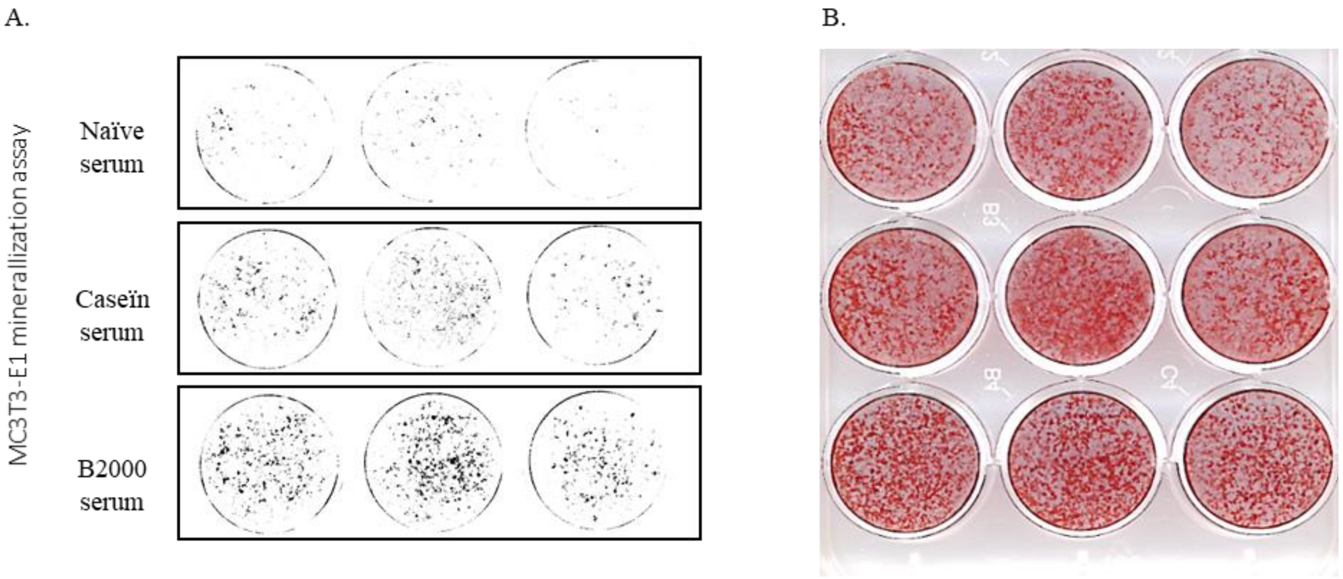

Figure S3. Alizarin red staining of the mineralization assay in MC3T3-E1 cells following enriched serum incubation (n = 3). Data supporting Figures 2D and 2E.

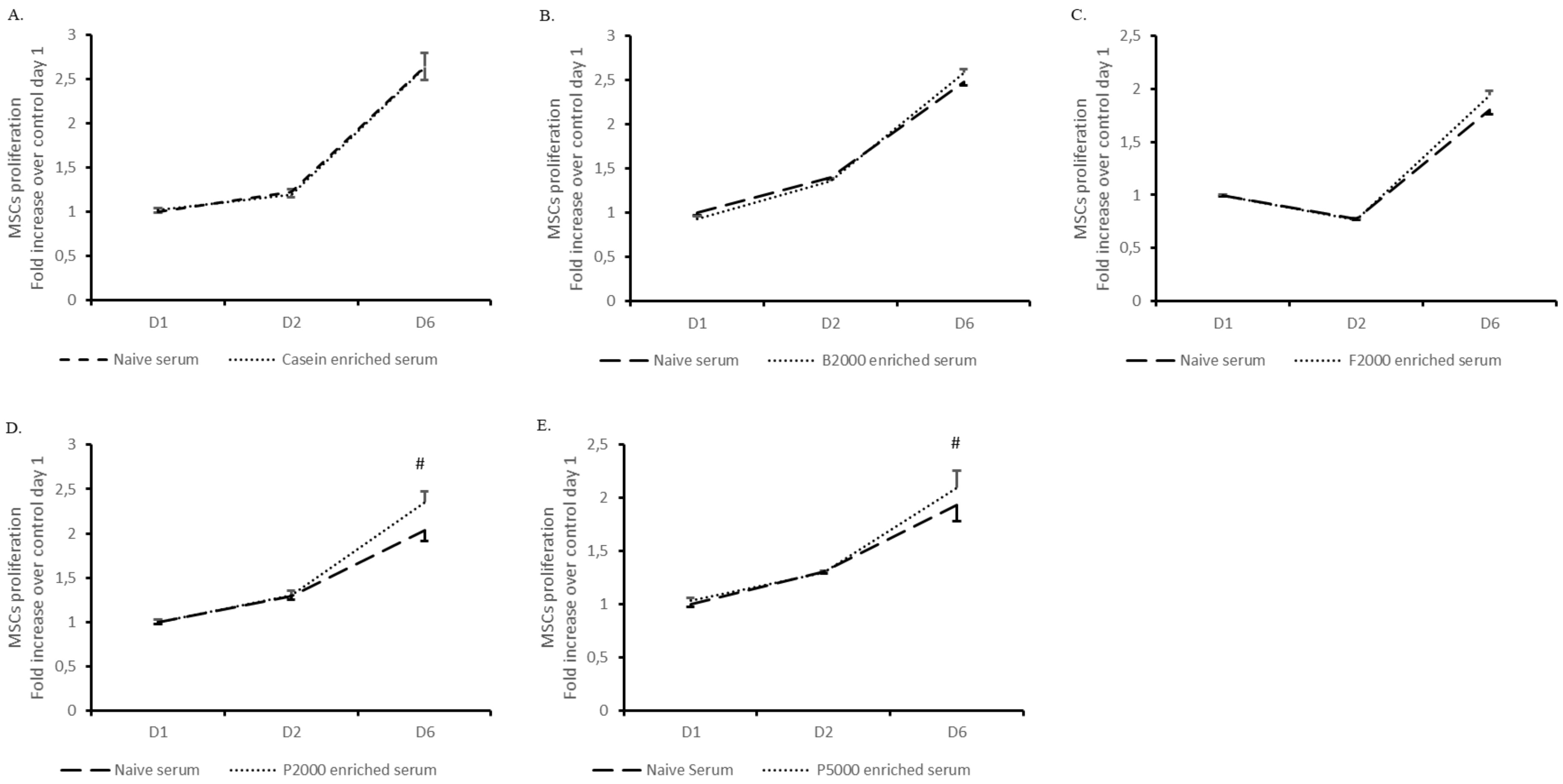

Figure S4. Human primary MSC proliferation from day 1 to day 6 following enriched serum incubation. This figure 5S further complements the figure 3B and the proliferation time course depending on protein origin **(A)** Hydrolysed casein; **(B)** B2000 (bovine, mean molecular weight 2kDa); **(C)** F2000 (fish, mean molecular weight 2kDa); **(D)** P2000 (porcine, mean molecular weight 2kDa) and (E) P5000 (porcine, mean molecular weight 5kDa). # $p < 0.05$

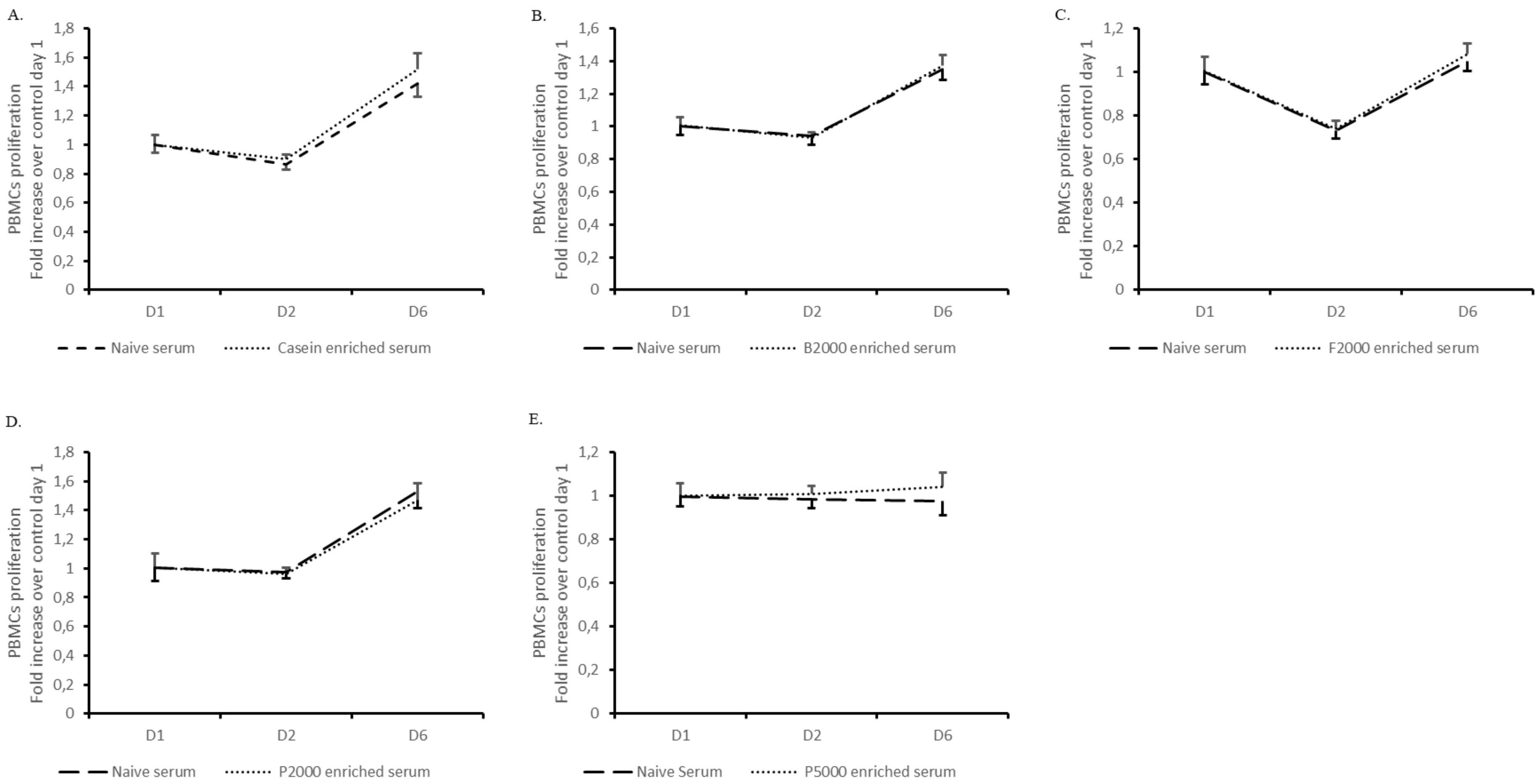

Figure S5. Human primary PBMC proliferation from day 1 to day 6 following enriched serum incubation. This figure 6S complements the figure 3C and the proliferation time course depending on protein origin **(A)** Hydrolysed casein; **(B)** B2000 (bovine, mean molecular weight 2kDa); **(C)** F2000 (fish, mean molecular weight 2kDa); **(D)** P2000 (porcine, mean molecular weight 2kDa) and **(E)** P5000 (porcine, mean molecular weight 5kDa).

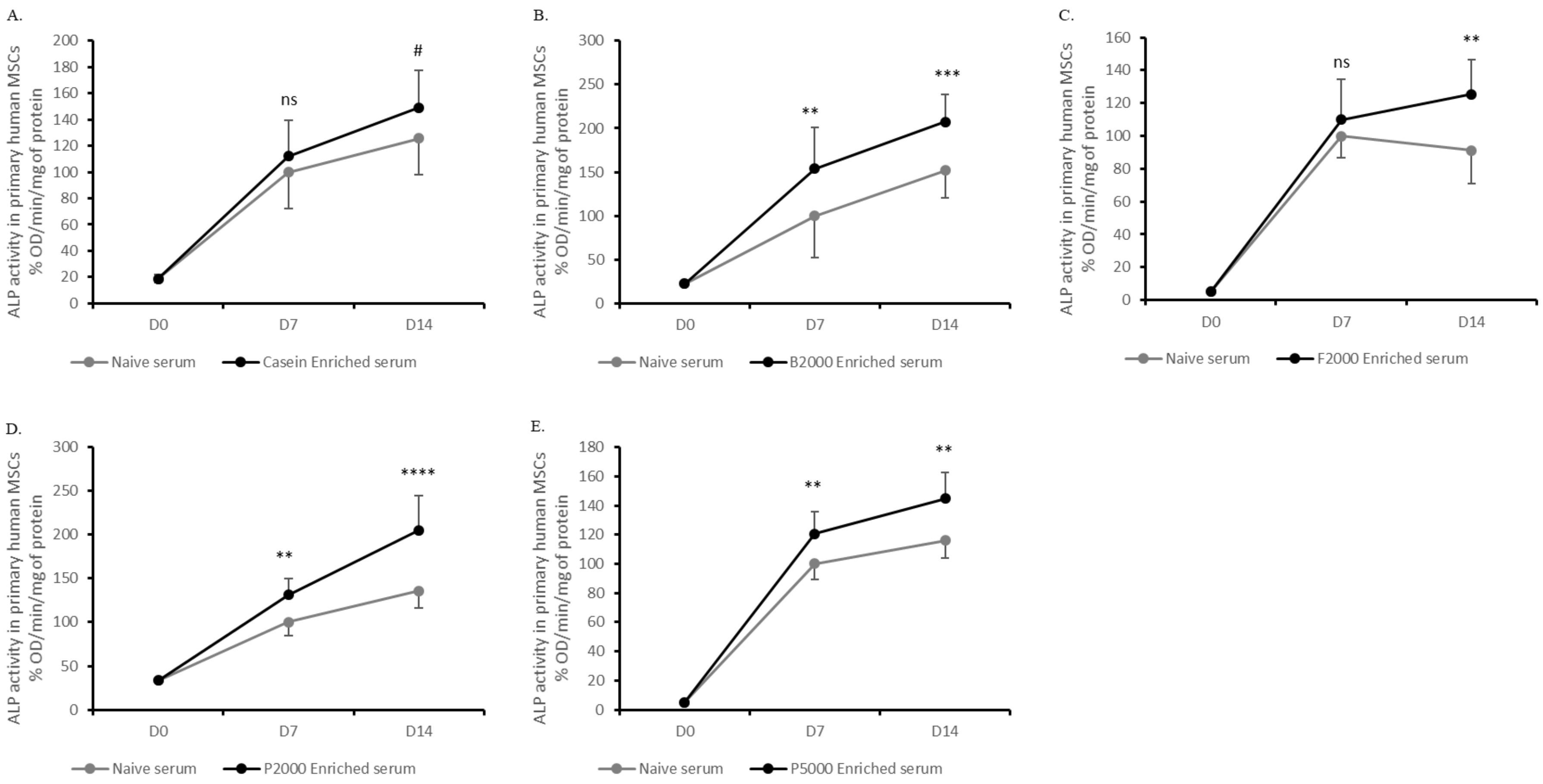

Figure 6. ALP activity of human primary MSCs from day 0 to day 14 following enriched serum incubation. This figure S6 complements the figure 4A and the ALP activity depending on incubation time and protein origin **(A)** Hydrolysed casein; **(B)** B2000 (bovine, mean molecular weight 2kDa); **(C)** F2000 (fish, mean molecular weight 2kDa); **(D)** P2000 (porcine, mean molecular weight 2kDa) and **(E)** P5000 (porcine, mean molecular weight 5kDa). # $p < 0.05$; * $p < 0.01$; ** $p < 0.001$; *** $p < 0.0001$; **** $p < 0.00001$.

Table S1. Composition of the diets: *in vivo* experiment (%).

Ingredients (%)	Control 15%	Control 17.5%	B2000
Casein	15	17.5	15
Sucrose	10	10	10
Dextrine	15.5	15.5	15.5
Cellulose	5	5	5
Saturated fat	2	2	2
Sunflower oil	2	2	2
Rape seed oil	3	3	3
B2000			2.5
L-cystine	0.18	0.18	0.18
Choline bitartrate	0.25	0.25	0.25
Mineral mix (AIN-93M-MX)	3.5	3.5	3.5
Vitamin mix (AIN-93-VX)	1	1	1
Corn starch	42.57	40.07	40.07
%	100	100	100
